# Assessment of the Knowledge and Practice of Infection Control among Radiographers in Saudi Arabia: A Cross-Sectional Survey Study

**DOI:** 10.3390/healthcare11212817

**Published:** 2023-10-24

**Authors:** Awadia Gareeballah, Samah Matar Al-sehli, Rana Theeb Al-mutairi, Moawia Gameraddin, Walaa Alsharif, Maisa Elzaki, Sultan Abdulwadoud Alshoabi, Kamal Dahan Alsultan, Amel F. Alzain, Awatif M. Omer, Zuhal Y. Hamd

**Affiliations:** 1 Department of Diagnostic Radiology Technology, College of Applied Medical Sciences, Taibah University, Al-Madinah Al-Munawwarrah 41477, Saudi Arabia; agsali@taibahu.edu.sa (A.G.); tu4052765@taibahu.edu.sa (S.M.A.-s.); tu4050672@taibahu.edu.sa (R.T.A.-m.); wsheref@taibahu.edu.sa (W.A.); mmohmmed@taibahu.edu.sa (M.E.); sshoabi@taibahu.edu.sa (S.A.A.); kdsultan@taibahu.edu.sa (K.D.A.); afzain@taibahu.edu.sa (A.F.A.); aiomer@taibahu.edu.sa (A.M.O.); 2 Department of Diagnostic Radiology, Faculty of Radiological Sciences and Medical Imaging, Alzaiem Alazhari University, Khartoum 13311, Sudan; 3 Department of Radiological Sciences, College of Health and Rehabilitation Sciences, Princes Nourah bint Abdulrahman University, Riyadh 11671, Saudi Arabia; zyhamd@pnu.edu.sa

**Keywords:** healthcare associated infections (HAIs), radiology departments (RD), personal protective equipment (PPE), professional experience, prior infection control training, sterilization, cleaning

## Abstract

Effective control of healthcare-associated infections (HAIs) involves a collaborative effort among various healthcare stakeholders, including healthcare workers, patients, and professionals. Radiographers, as essential members of the healthcare team, play a crucial role in HAI prevention by diligently adhering to standard infection control precautions (SICP) and maintaining a high level of knowledge regarding infection control procedures. The study aimed to assess the knowledge and practice of radiographers concerning infection control in radiology departments in Saudi Arabia. Methods: A descriptive cross-sectional study was conducted in Saudi Arabia in the period from February to May 2022, with data collected using an online survey in the form of a google forms questionnaire disseminated through social media as an electronic link and including the patient’s demographic characteristic such as age, gender, education level, experience, and prior infection control training and multiple closed ended questions to assess knowledge of standard infection control precautions and the practice of infection control. Overall, 113 participants responded to the survey and entered their responses directly, and the data were analyzed using the SPSS (statistical package for social science). Results: The study revealed that the mean score of knowledge and awareness of the practice of infection control among radiographers in Saudi Arabia was (63.0 and 61.9, respectively), which were considered moderate levels. Females were significantly more knowledgeable about infection control and more aware of the practice than males (*p*-values = 0.019). The participants who previously attended courses of infection control training had a significantly higher score with a mean rank of (60.9) than those who had not (43.4), (*p*-value = 0.013). The radiographers’ level of experience, age, and academic qualification had no significant influence on overall knowledge and practice of infection control (*p*-values > 0.05). Conclusions: In Saudi Arabia, radiographers have a moderate level of knowledge and practice of infection control. There is a need for an ongoing training and education program for practicing radiographers to ensure they perform better in infection control measures.

## 1. Introduction

Healthcare associated infections (HAIs) refers to infections acquired in any setting related to healthcare, including inpatient and outpatient facilities as well as radiology and emergency departments (RD and ED). Both patients and healthcare workers are impacted by healthcare-associated infections. Saudi Arabia is undergoing a major transformation as it strives to realize its ambitious “Vision 2030” program. In this context, an important goal of the ministry of health is to promote preventive care in order to address critical concerns such as HAIs and to implement proper infection control measures [1,2]. Radiographers, as healthcare workers, are thought to be at a significant risk of contracting and spreading diseases due to their close contact with patients and other hosts [3,4]. The radiology department faces two significant challenges when conducting routine examinations: one is to control cross-infection between patients, and the other is to protect staff from infection [5]. Radiographers must use standard infection control measures (SICP) to prevent HAIs. Radiographers’ responsibilities for related infection control include upholding a safe work environment, choosing suitable risk management and hazard control measures, and using reduction or elimination methods that adhere to health and safety regulations. Radiographers also have other responsibilities, such as applying the right disinfectant principles and applications, sterilization and decontamination procedures, and the precautions suggested for properly handling waste and spillage [4,6]. Several studies reported differences in infection control knowledge based on a team of healthcare workers and their experience, and other studies found variation in terms of understanding transmission and control of infection and its interpretation and implementation by healthcare workers [7,8,9,10].

The benefits of adequate infection control measures range from improved morbidity and mortality to disease transmission prevention and promoting cost-effective healthcare [11,12,13]. Implementing highly precautionary infection prevention and control measures in radiology can minimize the risk of spreading microbes to patients and radiographers. Radiographers need to be adequately knowledgeable about infection control precautions and best practices. Only a few studies in Saudi Arabia evaluate radiographers’ awareness of infection control. This study’s main goal was to evaluate the understanding and application of infection control among radiographers in the Kingdom of Saudi Arabia. In this study the knowledge of standard precautions and awareness of the practice among radiographers in Saudi Arabia were evaluated via an online questionnaire distributed through social media (WhatsApp’s—Facebook and twitter), and including the participants’ demographic data and multiple closed-ended questions related to knowledge of infection control, standard precautions, and awareness of the practice.

## 2. Materials and Methods

A cross-sectional study was carried out in Saudi Arabia in the period of February to May 2022, using an online questionnaire distributed among radiographers through social media. The study employed a non-probability sampling technique, specifically utilizing the Roasoft sample size calculator to determine the appropriate sample size based on the total number of radiographers in Saudi Arabia, which was determined to be 4669. The calculation was conducted using a 90% confidence interval (CI) and a 5% margin of error, resulting in a sample size of 256. The response rate for the study was 45.3%, with a total of 116 participants. However, it is noteworthy to mention that three participants were excluded from the final analysis as they declined to participate and complete the survey. A total of 113 participants agreed to participate and completed the survey. Ethical approval was obtained from the Research Ethics Committee of the College of Applied Medical Science, Taibah University, Madinah City, Western Region, KSA (Reference Number: 2021\125\312\DRD). Informed consent was obtained from all subjects prior to their voluntary participation in the study. The study questions were adapted from previous studies [14,15] and were modified to ensure their current suitability for the study participants. The modified version of the questionnaire was validated by experts, including three senior radiographers and two academic lecturers, to ensure that each question was suitable and precise. The validation of the questionnaire through pilot methods was not performed, the reason being that the questions in the questionnaire had already been validated face to face in previous studies from which the data collection sheet was prepared and, a pilot test was conducted and Cronbach’s alpha internal consistency coefficient was applied, the reliability score of the pilot test was found to be >0.7 [14,15]. Demographic background information, including gender, age, level of experience, and academic qualifications, were gathered in addition to the responses to the closed-ended questions dealing with knowledge and awareness of the practice of infection control among radiographers concerning infection control in radiology departments (“see Supplementary Materials”).

### 2.1. Data Analysis

The data were analyzed using IBM SPSS version 23 (IBM SPSS Statistics, Armond, NY, USA). The frequencies, mean, and standard deviations were calculated to describe the items. The radiographers’ knowledge and awareness of the practice of infection control in radiology departments were measured using 48 statements. The statements were dichotomized/classified into “correct” and “incorrect”, so the possible score ranged between zero (the least relevant to knowledge) and 48 (the most pertinent to the knowledge and awareness of the practice). Before going one step further for the analysis test, the normality was inspected, and the results of the Kolmogorov–Smirnov tests found that the distribution of the knowledge and awareness of the practice was not a normal distribution (*p*-value < 0.05); as a result, non-parametric tests were considered.

### 2.2. Non-Parametric Tests

As the data were not normally distributed, non-parametric tests were conducted (Mann–Whitney and Kruskal–Wallis) with a consideration of the post hoc (Dunn test) to test the multi-comparisons within the group. The level of knowledge and awareness of practice was inspected regarding socio-demographic factors; A *p*-value ≤ 0.05 was considered statistically significant.

## 3. Results

**Figure 1:**
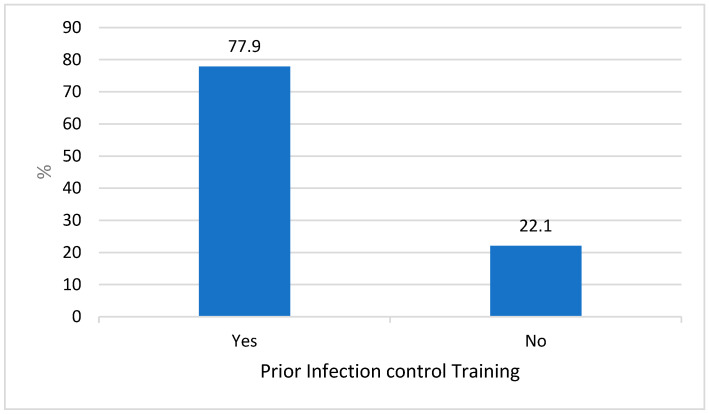
Prior training of infection control.

The result of the study demonstrated that about 77.9% of the study participants had prior infection control training (Figure 1). The demographic results of the study participants are listed in (Table 1).

Concerning knowledge of standard precautions, in this study, most radiographers were aware of the standard precautions designed for all healthcare personnel and patients (90.3%), as well as for all healthcare personnel and high-risk patients (58.4%). The majority of radiographers were knowledgeable about hand hygiene and aware that it is to be performed before and after contact with patients (89.4%), between patients (87.6%), after removing gloves (84.1%), and they also recognized that hand hygiene is the most effective way to limit disease transmission (84.1%) (Table 2).

**Figure 2:**
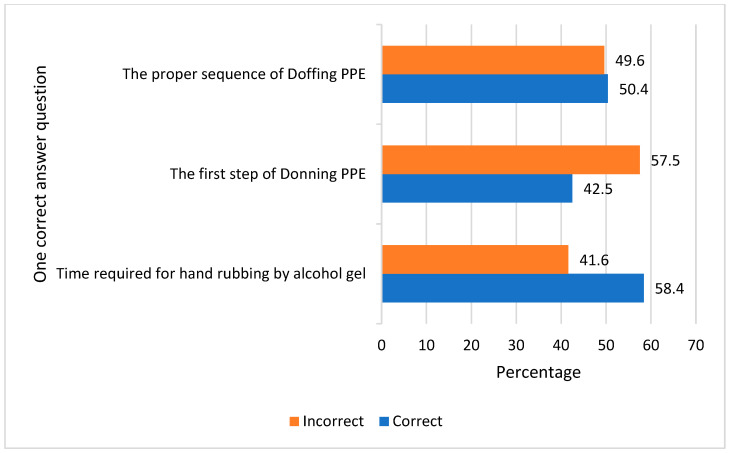
Radiographers’ knowledge of the standard precautions of infection control.

The study demonstrated that more than half of the study participants (54.9%) were knowledgeable of needle discarding without recapping in a specially designed bin, and only (31.0%) knew that alcohol gel only is not effective against spore microorganisms. Most responders (89.4%) identified all forms of PPE, and most of them described that during interventional radiology it is mandatory to use sterile gowns (82.3%). Most radiographers knew that masks (88.5%), eye googles (76.1%), and aprons (78.8%) are protective wear that is necessary to use when there is a risk of a splash of blood or body fluid (Table 2). Poor knowledge was found among radiographers concerning the first step of donning PPE, which was a gown (42.5%), and the proper sequences for doffing PPE, which were gloves, eye protection or face shield, gown, masks, or gloves, gown, eye protection, mask or respirator (50.4%) (Figure 2).

**Figure 3:**
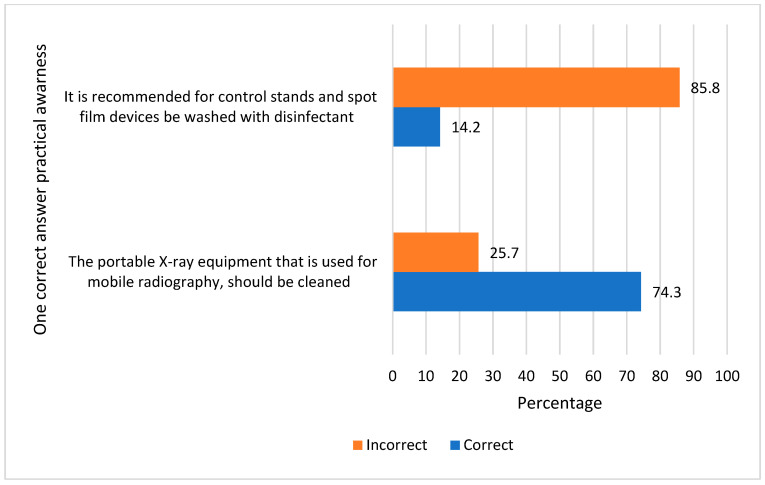
Radiographers’ awareness of the practice of infection control.

Concerning awareness of the practice of infection control, the study revealed that most of radiographer’s knowledge that the X-ray cassettes should be covered with plastic when examining patients in isolation (75.2%), 61.1% were aware that the washing and disinfection of anatomical markers should be performed weekly, and most of them had knowledge that workstations and procedure rooms should be cleaned each time they are used (83.2%). The vast majority of the radiographers (82.3%) were aware that all surfaces the patient might come into contact with should be cleaned and disinfected on a regular basis. The majority of the radiographers (80.5%) understood that plastic wrap must be changed between patients, and all headrests and sponge immobilizers must be covered. The majority of radiographers were aware that the shared X-ray equipment should be cleaned and disinfected with ethyl alcohol after each used (76.1%), and most of them (74.3%) knew that the mobile equipment is cleaned before and after procedures, whereas only 14.2% were aware that the spot film device requires weekly cleaning with disinfectant. (Table 3, Figure 3).

Table 4 summarizes the factors affecting the overall knowledge and awareness of the practice of infection control in terms of gender, age, education, experience, and prior infection control training. It was found that females were significantly more aware of knowledge and practice than males (mean rank 63.6 vs. 49.2, *p*-value = 0.019), radiographers who had prior infection control training were significantly more aware of the practice of infection control than those who had not (mean rank 60.9 vs. 43.4, *p*-value = 0.013). The radiographers’ level of experience, age, and academic qualification did not substantially affect their knowledge and awareness of infection control practices (*p*-value > 0.05). The study found that gender was an influencing factor for knowledge of precautions; the score of knowledge was significantly higher in females than in males (*p*-value < 0.05). Meanwhile, age and prior control training were two factors that also significantly influenced the level of awareness of the practice of infection control (*p*-value < 0.05 and 0.01, respectively), (Table 5).

## 4. Discussion

The goal of infection prevention and control (IPC) strategies is to stop and limit the transmission of diseases among individuals in healthcare environments. The danger of transmitting germs to patients and radiographers can be reduced by using extremely cautious infection prevention and control methods in radiology. The current study assessed the knowledge and awareness of the practice of infection prevention and control among radiographers.

The present study found that the score of knowledge of infection control was acceptable among Saudi radiographers. Several studies have been conducted to assess radiographers’ knowledge and practices of infection control, and discrepancies have been reported in their knowledge levels [16,17,18]. A study conducted in Palestine by Alnnahal et al. demonstrated moderate knowledge and practice of infection control among Palestinian radiographers [19]. Similarly, a study conducted in Jordan by Abdelrahman et al. reported a moderate level of knowledge among Jordanian radiographers regarding nosocomial infection control practices [14]. Another study in Malawi by Nyirenda et al. found that radiographers had moderate practice in and knowledge of infection control procedures [20]. Ahmed et al. demonstrated good knowledge [21]. Aljunaidi et al. and Almatari et al. conducted a study to assess the knowledge and practice of infection control in COVID-19, and both reported good knowledge of guidelines for infection control. On the other hand, Almatari et al. found a need for more knowledge on the practical side [22,23]. These findings highlighted the need for improved awareness and education among radiographers about infection transmission routes and proper infection control procedures.

The study found that about two-thirds of participants understood infection control standard precautions. These precautions include using the proper personal protection equipment while interacting with a patient and his products and practicing good hand hygiene. This rate, however, is still greater than those found in studies conducted in a hospital in Northern Cyprus [24], among nursing students in Jordan (49.64%) [25], and among dentistry faculty members and students (third to fifth-year) in Riyadh, KSA (49–49.6%) [26]. Studies conducted in Iran [27] and Nigeria [28] showed gaps in HCWs’ awareness of basic precautions. Because of the knowledge gap among HCWs, more emphasis on infection control standard precautions should be placed in academic and continuing professional development training courses.

The study assessed the factors affecting the knowledge of infection control in terms of gender, age, education, experience, and prior infection control training. It was found that females were significantly more knowledgeable than males, and those with prior training in infection control were more aware of infection control and practice. Consistently, according to Abalkhail et al., training and prior exposure to HAIs were significant predictors of excellent practice [13]. On the other hand, females were significantly more aware of the practice of infection control than males. In contrast, a previous study reported that the female sex was negatively linked with knowledge and practice [28]. Knowledge is pivotal to developing good knowledge of practice; therefore, it is a significant motivator to introduce positive change in practice [13,23].

Most radiographers were knowledgeable about proper hand hygiene before and after working with patients, cleaning radiographic equipment, adhering to standard precautions, and thinking that standard precautions stop the spread of infections. This awareness is likely because these procedures became standard operating procedures supported by the institution’s firm institutional culture and the infection control regulations adopted by the Ministry of Health, KSA [13].

Most radiographers had knowledge of all forms of PPE, but less than half of them were aware of the first step of donning and the sequence of doffing PPE. Another study by Ashoor et al. showed the inadequate practice of donning and doffing PPE among healthcare workers in eastern Saudi Arabia during the early stages of the COVID-19 pandemic [29].

There was some confusion concerning the appropriate PPE that was to be used during the interventional procedures: in addition to the sterile gown that was chosen by 82.3% of the radiographers, approximately 91.2% chose sterile gloves, and 98.2% chose protective masks. This finding is consistent with a study that was carried out among Jordanian radiographers [14]. Radiographers must wear sterile gowns daily as they perform or assist with invasive procedures. For vascular compression, filling ultrasound gel containers, or when there is a risk of coming into contact with blood and other bodily fluids, clean disposable gloves should be worn. In situations where there is a chance of splashes, protective eye goggles and an apron should also be worn [14]. Additionally, gloves must be worn whenever the radiographer comes into contact with a patient’s mucous membranes or non-intact skin, when examining a patient infected with bacteria that are resistant to antibiotics and when handling contaminated objects like soiled linens, and whenever the radiographer has a cut on their hands. On the other hand, sterile gloves should only be worn by radiographers when they are involved in the sterile field during invasive procedures [14,30]. These results emphasize the need for education and training on the proper use and selection of PPE to ensure that healthcare professionals are equipped with the knowledge and skills to protect themselves and their patients.

Over fifty percent of radiographers were uncertain about the appropriate course of action regarding a used needle. They should select to recap the needle with care and store it in the patient’s room. One study demonstrated that radiologic technologists and radiology trainees sustained the highest number of injuries. The primary reason for injury was the insertion of a cannula; however, numerous injuries occurred while discarding items or manipulating the waste receptacle [31]. A Nigerian study showed that radiographers lacked the knowledge that a recapped needle poses a potential risk of infection [32].

In terms of awareness of practice, in this study, it was found that most radiographers were aware that X-ray cassettes are covered with plastic when examining isolated patients. Also, the responding radiographers were aware of the practice of cleaning and sterilizing the anatomical marker and lead rubber apron with an antiseptic solution once a week, as well as disinfecting all workstations and procedure rooms used between each use (61.1%, 83.2%, respectively). Nyirenda D et al., found that 92% of the respondents to the study always cover the X-ray cassette with plastic when examining isolated patients, while only 10% cleaned and sterilize the anatomical marker and lead rubber apron with a disinfectant solution every week [20].

Radiographers are generally aware of the importance of wearing protective equipment and never entering the sterile area during a sterile procedure. However, only 23% of them were aware that the back of the sterile gown is non-sterile and should not come into contact with the field. Additionally, 17.7% of radiographers know that scrub personnel must remain in the operation room when multiple images are taken during a sterile procedure.

There were no significant differences in overall knowledge and practice scores, radiographer age range, duration of experience, and academic qualification. This result is not surprising since a study among Jordanian radiographers found that knowledge levels were unaffected by experience or age [14]. Furthermore, according to another study conducted by Slater et al., which included “Australian nurses from five different hospital departments, age, duration of experience”, and academic qualification had no significant impact on infection, prevention, and control. Additionally, in a study on Saudi radiology professionals, staff with more than ten years of experience were much more likely to practice infection control [33,34]. Another study conducted by Alnahhal et al. reported that age and professional experiences were two factors that significantly impact levels of knowledge and practice of infection control among radiographers at governmental hospitals in Palestine [19]. NAJI et al. found inadequate knowledge and practice in infection prevention and control, respectively among Yemeni radiographers; age, educational status, and occupational experience significantly influenced the knowledge, and practice scores, while gender showed no significant influence [35].

The study challenged several limitations, including a smaller sample size, and this might be owing to the time constrains of the study. In addition, despite the team’s research efforts, the study questionnaire failed to garner the required sample size. This challenge may be due to several reasons, such as survey fatigue, lack of motivation, participants not actively engaging with online platforms, and being too busy. As a questionnaire was used in this study, there was a possibility of bias as participants could have selected random responses without reading the entire questionnaire. Another limitation of this study is that it evaluated the reported practice, which is based on radiographers self-reporting and may not reflect the actual practice. These limitations did not influence the whole results, which were consistent with previous studies. Infection control guidelines should be included in the radiology curriculum and ensure that the student radiographers know the guidelines before graduation; this can contribute to patient care and safety. It is also suggested that the hospital administration develop an environment suitable for its implementation, such as making infection control courses mandatory throughout the internship year. In future studies, it is suggested that a large sample size be used; the larger sample size can improve precision and generalizability and allow for more robust conclusions and recommendations from the study results. Using interviews as a research method in future studies can also provide valuable insights. Interviews allow for in-depth exploration of participants’ perspectives and experiences, which can help deliver more accurate and bias-free results.

## 5. Conclusions

This study concluded that the radiographers showed average knowledge and practice of infection control. Further, the study found that prior infection control training is significantly correlated with the best knowledge and practice. Therefore, setting up training programs for radiographers may help revive and enhance their understanding of standard infection control precautions and is also anticipated to enable good practice. There was no association between professional experience and education level with overall knowledge and awareness of the practice of infection control, suggesting that earlier academic curricula may have needed to have effectively covered subjects on infection control in healthcare settings.

## Figures and Tables

**Table 1:** Demographic characteristics of the study population.

	Variables	Frequency	Percentage
**Gender**	Female	61	54.0
	Male	52	46.0
**Age groups**	<26	51	45.1
	26–30	34	30.1
	31–40	18	15.9
	>41	10	8.8
	1–5	74	65.5
**Professional experience (Years)**	6–10	19	16.8
	11–15	8	7.1
	16–20	5	4.4
	>20	7	6.2
**Field of work**	Conventional radiology	39	34.5
	Ultrasound	17	15.0
	Interventional radiology	5	4.4
	Computerized tomography (CT)	25	22.1
	Magnetic resonance imaging (MRI)	22	19.5
	Nuclear medicine	5	4.4
**Education level**	Diploma	7	6.2
	Bachelor’s	87	77.0
	Master’s	16	14.2
	Ph.D.	3	2.7

**Table 2:** Radiographers’ knowledge of standard precautions concerning infection control in radiology departments.

Statements	Correct Answer	Correct Response %
**Policy and procedures for infection control precautions designed for?**		
Only healthcare professionals	No	49.6
All healthcare professionals and patients at high risk	Yes	58.4
All patients and healthcare professionals	Yes	90.3
2. **Concerning the Hand hygiene**		
done before coming into contact with a patient	Yes	89.4
carried out following contact with a patient	Yes	89.4
a procedure is done between two patients	Yes	87.6
carried out after taking off the gloves.	Yes	84.1
Is the most practical way to stop the spread of disease	Yes	84.1
3. **After the use of a needles**		
After use, place it on a tray in the patient’s room.	No	53.1
after usage, recap with caution	No	31.9
without recapping, discarded in a designed bin.	Yes	54.9
4. **Rubber or latex gloves must be worn by the radiographer.**		
For each method/procedure	No	19.5
When there is a risk arising from contact with blood	Yes	92
When there is a risk of a cut	Yes	73.5
If there is a skin cut	Yes	82.3
5. **In case of spray of blood or body fluids, the radiographer ought to wear**		
A mask	Yes	88.5
Eye goggles	Yes	76.1
An apron	Yes	78.8
Overshoes	No	10.6
6. **What is the mandatory protective wear for the radiographer use at all times when doing interventions?**		
Sterile gloves	No	8.8
Sterile gown	Yes	82.3
Protective mask	No	1.8
7. **PPE includes items like gloves, isolation gowns, face masks, particulate respirators, and eye protection**.	Yes	89.4
8. **Is Alcohol gel alone effective against spore-forming organisms?**	No	31.0

The score for knowledge Keys: 0–13 Low, Moderate 14–21, 22–27 = High. The total score for knowledge of precaution = 17 (63.0).

**Table 3:** Awareness of the practice of infection control in radiology departments.

Statements	Correct Answer	Correct Responses %
**Radiographers must follow the following guidelines to prevent disease transmission within the radiology department:**		
Spills of bodily fluid should never be cleaned up by the radiographer; always let the cleaning staff handle it.	No	42.5
Excretions or secretions from patients can be disposed of in sinks.	No	19.5
The plastic wrap must be changed between each patient, and all headrests and sponge immobilizers must be covered.	Yes	80.5
After each procedure, the radiographer must clean the radiographic tables.	Yes	85.0
Patients who are excessively coughing in the waiting area need to be put in a solitary room as soon as feasible.	Yes	82.3
2. **Which of the following statements about sterilizing the radiography equipment is true?**		
The duty to ensure that any radiographic equipment used during a sterile treatment is clean rests with the nurse, not the radiographer.	No	45.1
Cleaning with a disinfectant solution is necessary for image receptors, portable radiography devices, and overhead units.	Yes	85.0
3. **When moving around during a sterile procedure, which of the following considerations must the radiographer keep in mind?**		
Never should he/she reach across a sterile field.	Yes	62.8
He or she may turn away from the barren field.	No	23.9
They must proceed back-to-back if they need to pass another sterile person.	Yes	61.9
The radiographer might give the scrub nurse the image receptor to hold in their direction.	Yes	57.5
Scrubbed staff must, whenever feasible, leave the operating room if numerous radiographs are to be obtained.	No	17.7
4. **When investigating patients in isolation, are X-ray cassettes protected with plastic?**	Yes	75.2
5. **How often should the lead rubber apron and anatomical marker be cleaned and sanitized with the antiseptic solution?**	Yes	61.1
6. **Each time they are used, all workstations and process rooms should be cleaned and disinfected.**	Yes	83.2
7. **Any single-use tools or covers that were used during procedures should be thrown away right away.**	Yes	72.6
8. **Using a portable US and a C-arm, central venous catheter (CVC) installation is carried out in an isolation room.**	Yes	57.5
9. **All surfaces the patient might come into contact with should be cleaned and disinfected on a regular basis.**	Yes	82.3
10. **When using shared equipment, disinfect it with 70% ethyl alcohol after each use.**	Yes	76.1

The score for awareness of practice Keys: 0–11 low, moderate 12–16, 17–21 = high. The score for overall knowledge and practice Keys: 0–24 low, 25–38 moderate, 39–48 high. The score for awareness of the practice of infection control = 13 (61.9). Overall knowledge and awareness of the practice of infection control= 30 (62.5).

**Table 4:** Factors affecting the overall score of knowledge and awareness of the practice of infection control.

Factor		Knowledge of Infection Control		
		Mean Rank	Statics	*p*-Value
**Gender**	Female	63.6	1239	0.019 *
	Male	49.2		
**Age**	<26	51.0	5.164 (ns)	0.160
	26–30	65.8		
	31–40	52.7		
	>41	65.4		
**Professional experience (years)**	1–5	55.7	1.209 (ns)	0.877
	6–10	57.5		
	11–15	68.9		
	16–20	57.5		
	>20	55.9		
**Education**	Diploma	48.6	2.911 (ns)	0.406
	Bachelor’s	56.1		
	Master’s	60.1		
	Ph.D.	85.2		
**Prior infection control training**	Yes	60.9	1077	0.013 *
	No	43.4		

* ≤ 0.05; ns = Not significant.

**Table 5:** Factors affecting knowledge and awareness of infection control.

Factor		Knowledge of Precaution			Practice Awareness		
		Mean Rank	Statics	*p*-Value	Mean Rank	Statics	*p*-Value
**Gender**	Female	63.8	1196 *	0.016	59.6	1412	0.313
	Male	49.0			53.7		
**Age**	<26	54.7	0.561 (ns)	0.905	50.0	9.040 *	0.029
	26–30	59.0			68.3		
	31–40	57.5			48.7		
	>41	61.2			69.2		
**Professional experience (years)**	1–5	57.1	0.948 (ns)	0.918	55.3	2.239 (ns)	0.692
	6–10	59.5			54.4		
	11–15	61.1			71.1		
	16–20	54.5			60.2		
	>20	46.9			64.2		
**Education**	Diploma	50.2	1.225 (ns)	0.747	53.9	2.032 (ns)	0.566
	Bachelor’s	57.3			55.4		
	Master’s	55.0			63.3		
	Ph.D.	74.3			77.2		
**Prior infection control training**	Yes	57.8	1029 (ns)	0.621	61.3	719.0 **	0.008
	No	54.2			41.8		

* ≤ 0.05; ** ≤ 0.01; ns = Not significant.

## Data Availability

The data presented in this study are available on request from the corresponding author.

## Supplementary Materials

The following supporting information can be downloaded at: https://www.mdpi.com/article/10.3390/healthcare11212817/s1.

## Abbreviations

CT	Computerized Tomography
ED	Emergency Departments
MRI	Magnetic Resonance Imaging
RD	Radiology Departments
HAIs	Healthcare Associated Infections
SCIP	Standard Infection Control Precautions
PPE	Personal Protective Equipment
