# Supplementary material for: Assessment of the Knowledge and Practice of Infection Control among Radiographers in Saudi Arabia: A Cross-Sectional Survey Study
Source: Healthcare (Basel). 2023 Oct 24;11(21):2817. doi: 10.3390/healthcare11212817 (PMC10648768; doi:10.3390/healthcare11212817)
Supplement: Supplementary file 1 [file healthcare-11-02817-s001.zip › healthcare-2585449-supplementary.pdf]

Assessment of Knowledge and Practices of Infection Control Among Radiographers in Saudi Arabia: A Cross-sectional Survey Study

Part one : Demographic informations

**Categories**	Answers					
**Age / years**	<26	26-30	31-40	> 40		
**Gender**	Male	Female				
**Professional experiences / years**	1-5	6-10	11-15	16-20	> 40	
**Field of works**	Conventional radiology	Ultrasound	Interventional radiology	CT	MRI	Nuclear medicine
**Academic degree**	Diploma's	Bachelor's	Master's	Ph.D.		
**Prior infection control training**	Yes	No				

Part two: Knowledge of the standard precautions of infection control

**Statements**	**Responses and answers**		
	Yes	No	Ido not know
**1. Policy and procedures for infection control precautions designed for?**			
Only healthcare professionals			
All healthcare professionals and patients at high risk			
All patients and healthcare professionals			
**2. Concerning the Hand hygiene**			
done before coming into contact with a patient			
carried out following contact with a patient			
a procedure is done between two patients			
carried out after taking off the gloves.			
Is the most practical way to stop the spread of disease			
**3. After the use of a needles**			
After use, place it on a tray in the patient's room.			
after usage, recap with caution			
without recapping, discarded in a designed bin.			
**4. Rubber or latex gloves must be worn by the radiographer.**			
For each method/procedure			
When there is a risk arising from contact with blood			
When there is a risk of a cut			
If there is a skin cut			
**5. In case of spray of blood or body fluids, the radiographer ought to wear**			
A mask			
Eye goggles			
An apron			
Overshoes			
**6. What is the mandatory protective wear for the radiographer use at all times when doing interventions?**			
Sterile gloves			
Sterile gown			
Protective mask			
**7. PPE includes items like gloves, isolation gowns, face masks, particulate respirators, and eye protection**.			
**8. Is Alcohol gel alone effective against spore-forming organisms?**			

9. What is the first step of Donning PPE?

a. Put on a mask ( ) b. Put on a gown ( ) c. Put on an eye protection Put on a gloves ( )

10. What is the right sequence of Doffing PPE?

a. Remove gown, mask, eye protection, gloves ( ) b. Remove gloves, gown, eye protection, mask ( ) c. Remove eye protection, mask, gown, gloves ( ) d. Remove gloves , eye protection, gown , mask **( )**

11. When using Alcohol-based hand rub technique for hand hygiene, what the time should be spent in hand rubbing?

a. 20-30 second ( ) b. 10-20 second ( ) c. Am not sure ( ) d. Idonot know ( )

Part three: Awareness' of the practice of infection control

1. **The portable x-ray equipment that used for mobile radiography, should be cleaned**

a. before procedure ( ) b. after procedure ( ) c. before and after procedure ( )

d. I'm not sure ( ) e. I don't know ( )

2. **It is recommended for control stands and spot film devices to be washed with disinfectant:**

b. Daily ( ) b. Weekly ( ) c. Monthly ( ) d. I'm not sure ( ) e. I don't know ( )

Statements	Responses and answers		
	Yes	No	Ido not know
**3. Radiographers must follow the following guidelines to prevent disease transmission within the radiology department:**			
Spills of bodily fluid should never be cleaned up by the radiographer; always let the cleaning staff handle it.			
Excretions or secretions from patients can be disposed of in sinks.			
The plastic wrap must be changed between each patient, and all headrests and sponge immobilizers must be covered.			
After each procedure, the radiographer must clean the radiographic tables.			
Patients who are excessively coughing in the waiting area need to be put in a solitary room as soon as feasible.			
**4. Which of the following statements about sterilizing the radiography equipment is true?**			
The duty to ensure that any radiographic equipment used during a sterile treatment is clean rests with the nurse, not the radiographer.			
Cleaning with a disinfectant solution is necessary for image receptors, portable radiography devices, and overhead units.			
**5. When moving around during a sterile procedure, which of the following considerations must the radiographer keep in mind?**			
Never should he/she reach across a sterile field.			
He or she may turn away from the barren field.			
They must proceed back-to-back if they need to pass another sterile person.			
The radiographer might give the scrub nurse the image receptor to hold in their direction.			
Scrubbed staff must, whenever feasible, leave the operating room if numerous radiographs are to be obtained.			
**6. When investigating patients in isolation, are X-ray cassettes protected with plastic?**			
**7. The lead rubber apron and anatomical marker be cleaned and sanitized with the antiseptic solution weekly?**			
**8. Each time they are used, all workstations and process rooms should be cleaned and disinfected.**			
**9. Any single-use tools or covers that were used during procedures should be thrown away right away.**			
**10. Using a portable US and a C-arm, central venous catheter (CVC) installation is carried out in an isolation room.**			
**11. All surfaces the patient might come into contact with should be cleaned and disinfected on a regular basis.**			
**12. When using shared equipment, disinfect it with 70% ethyl alcohol after each use.**			
